# What do Cochrane systematic reviews say about the management of irritable bowel syndrome?

**DOI:** 10.1590/1516-3180.2018.053740119

**Published:** 2019-05-08

**Authors:** Rafael Leite Pacheco, Arnaldo Roizenblatt, Aécio Flávio Teixeira de Góis, Carolina de Oliveira Cruz Latorraca, Carolina Frade Magalhães Girardin Pimentel Mota, Rachel Riera

**Affiliations:** I MD. Postgraduate Student, Evidence-Based Health Program, Universidade Federal de São Paulo (UNIFESP), and Assistant Researcher, Cochrane Brazil, São Paulo (SP), Brazil.; II Undergraduate Medical Student, Escola Paulista de Medicina (EPM), Universidade Federal de São Paulo (UNIFESP), São Paulo (SP), Brazil.; III MD, MSc, PhD. Cardiologist and Adjunct Professor, Discipline of Evidence-Based Medicine, Escola Paulista de Medicina (EPM), Universidade Federal de São Paulo (UNIFESP), São Paulo (SP), Brazil.; IV MSc. Psychologist and Postgraduate Student, Evidence-Based Health Program, Universidade Federal de São Paulo (UNIFESP), and Assistant Researcher, Cochrane Brazil, São Paulo (SP), Brazil.; V MD, MSc, PhD. Gastroenterologist and Adjunct Professor, Discipline of Evidence-Based Medicine, Escola Paulista de Medicina (EPM), Universidade Federal de São Paulo (UNIFESP), São Paulo, Brazil.; VI MD, MSc, PhD. Rheumatologist and Adjunct Professor, Discipline of Evidence-Based Medicine, Escola Paulista de Medicina (EPM), Universidade Federal de São Paulo (UNIFESP), and Researcher, Cochrane Brazil, São Paulo (SP), Brazil.

**Keywords:** Review [publication type], Irritable bowel syndrome, Colonic diseases, functional, Evidence-based medicine, Evidence-based practice

## Abstract

**BACKGROUND:**

Irritable bowel syndrome (IBS) is a clinical disorder associated with high socioeconomic burden. Despite its importance, management of IBS remains difficult and several interventions have been hypothesized as beneficial for this condition. This study identified and summarized all Cochrane systematic reviews (SRs) about the effects of interventions for managing IBS patients.

**DESIGN AND SETTING:**

Review of systematic reviews, carried out in the Discipline of Evidence-Based Medicine, Escola Paulista de Medicina (EPM), Universidade Federal de São Paulo (UNIFESP).

**METHODS:**

Review of Cochrane SRs addressing interventions for IBS.

**RESULTS:**

We included six SRs assessing acupuncture, bulking agents, antispasmodics, antidepressants, herbal medicines, homeopathy, hypnotherapy and psychological therapy for IBS. The certainty of evidence ranged from unknown to moderate, mainly due to imprecision in the estimates and high risk of bias from the primary studies included. There was moderate certainty of evidence that acupuncture had no important benefit regarding improvement of symptoms and quality of life, compared with sham acupuncture. There was also very low certainty of evidence that homeopathic asafoetida, used alone or in association with nux, was better than placebo regarding self-reported overall improvement.

**CONCLUSION:**

There was moderate certainty of evidence that acupuncture had no important benefit regarding improvement of symptoms and quality of life. Further well-designed and well-conducted randomized clinical trials are needed in order to reduce the uncertainties regarding the most commonly used interventions for patients with IBS.

## INTRODUCTION

Irritable bowel syndrome (IBS) is a frequent clinical disorder. Its prevalence has been estimated as 11% worldwide,^1^ with a range from 3% to 15% according to the diagnostic criteria used.^2^ The definition most commonly used is the one that was proposed by the Rome IV investigators, and this takes into account recurrent abdominal pain associated with other gastrointestinal symptoms, without a clear organic cause.^3^ The classification systems used generally envisage three groups: IBS predominantly involving constipation, IBS predominantly involving diarrhea and mixed IBS.^3^

Although IBS is a common disease, the etiological and pathophysiological aspects of this condition remain unclear and a matter of controversy. In the past, it was hypothesized that IBS might be more commonly associated with other frequently observed conditions such as sleep disorders and psychological disorders, and that these could be considered to be triggers of this disorder.^4^ Recent studies have suggested that the pathophysiology of this so-called brain-gut disorder is more complex and that it involves neurohormonal deregulation, bacterial overgrowth, food intolerance, inflammation, altered intestinal barriers, alterations to fecal flora, and genetic influence. This myriad of factors has been transforming recent knowledge of IBS.^5^

IBS gives rise to an important socioeconomic burden, due to its high prevalence and its impact on daily activities.In fact, occurrences of IBS have been correlated with considerable levels of healthcare demand and missing work days, thereby contributing towards higher direct and indirect costs in a variety of healthcare systems.^6^^,^^7^^,^^8^

Management of IBS remains a challenge. Several interventions have been used in clinical practice, including pharmacological, psychological, behavioral and complementary interventions.^9^ In some cases, practical recommendations are made on the basis of low levels of clinical evidence, relating only to hypothesized aspects of the pathophysiology of the condition.^9^

Since IBS is a highly prevalent condition associated with a heavy socioeconomic burden, systematic reviews addressing interventions for treating this condition are needed in order to guide decision-making. Cochrane systematic reviews are considered to provide reliable evidence and are a useful tool for healthcare providers and patients.

## OBJECTIVE

To summarize and present the evidence from Cochrane systematic reviews assessing interventions for management of irritable bowel syndrome patients.

## METHODS

### Design and setting

This was a review of Cochrane systematic reviews (SRs) carried out in the Discipline of Evidence-based Medicine of Escola Paulista de Medicina (EPM), Universidade Federal de São Paulo (UNIFESP). This manuscript was prepared for the section “Cochrane Highlights” of the São Paulo Medical Journal. It forms part of a formal collaboration between the São Paulo Medical Journal and the Cochrane Collaboration, and it is supported by Cochrane Brazil. The aim of this initiative is to disseminate the evidence from Cochrane SRs.

### Inclusion criteria

#### Types of studies

We included only the latest published version of Cochrane systematic reviews (SRs). We excluded all protocols, or any SR marked as “withdrawn” in the Cochrane Database of Systematic Reviews (CDSR).

#### Types of participants

We considered any participant who had been diagnosed with irritable bowel syndrome, as determined through the criteria of the original review authors. Reviews addressing irritable bowel syndrome and also other clinical situations were included only if the subset of data relating to irritable bowel syndrome participants was provided separately.

#### Types of intervention

We considered any pharmacological or non-pharmacological intervention for therapeutic purposes, compared with placebo, no intervention or any other intervention.

#### Type of outcomes

We considered the clinical and laboratory outcomes that had already been considered by the SR authors. When multiple outcomes were presented, we chose the primary safety and effectiveness outcomes or the most clinically relevant outcomes, to present in the current review.

### Search for reviews

We performed a systematic search in the Cochrane Database of Systematic Reviews (via Wiley) on December 4, 2018. The search strategy is fully depicted in Table 1.

**Table 1.** Search strategy

#1 MeSH descriptor: [Irritable Bowel Syndrome] explode all trees
#2 (Syndrome, Irritable Bowel) or (Syndromes, Irritable Bowel) or (Colon, Irritable) or (Mucous Colitides) or (Colitis, Mucous) or (Irritable Bowel Syndromes) or (Mucous Colitis) or (Irritable Colon) or (Colitides, Mucous)
#3 #1 or #2
Filters: in Cochrane Reviews; *in Title, Abstract, Keywords*

### Selection of systematic reviews

The selection process was performed by two authors, who independently screened all titles and abstracts retrieved through the electronic search. The authors checked whether the abstracts thus retrieved fulfilled the inclusion criteria and decided whether to include or exclude them. Any disagreements in the selection process were resolved through reaching a consensus.

### Presentation of the results

We produced a synthesis and presented the following characteristics relating to the reviews that were included: PICOs (population, intervention, comparator and outcomes), objectives, methods, main results, risk of bias from the original studies and certainty of evidence through the GRADE approach;^10^ along with the conclusions from the authors of the SRs that were included.

## RESULTS

### Search results

The initial search retrieved 78 abstracts of systematic reviews (SRs). After the selection process, six SRs were found to fulfill our inclusion criteria and were included in the analysis.^11^^,^^12^^,^^13^^,^^14^^,^^15^^,^^16^

### Results from systematic reviews

The six SRs included assessed the effects of conventional interventions (bulking agents, antispasmodics and antidepressants) and non-conventional interventions (acupuncture, herbal medicines, homeopathy, psychological therapy and hypnotherapy) for participants with irritable bowel syndrome (IBS). The main findings from the SRs included, and the quality of the evidence (based on the GRADE approach),^10^ are detailed in Table 2. A brief summary of each SR is presented below.

**Table 2.** Characteristics, main results and quality of evidence of the systematic reviews included

Intervention	Comparison	Main findings	Evidence certainty (GRADE)*
Acupuncture	Acupuncture versus sham	*No difference* • Symptom severity • Quality of life	Moderate Moderate
	Acupuncture versus pharmacological treatment	*Benefits* • Proportion of participants with symptom improvement	Moderate
Antispasmodic drugs	Antispasmodic drugs versus placebo	*Benefits* • Improvement of abdominal pain • Overall assessment • Symptom score	NA NA NA
Antidepressants	Antidepressants versus placebo	*Benefits* • Improvement of abdominal pain • Overall assessment • Symptom score	NA NA NA
Bulking agents	Bulking agents versus placebo	*No difference* • Improvement of abdominal pain • Overall assessment • Symptom score	NA NA NA
Herbal medicines	Standard Chinese herbal formulation versusplacebo	*Benefits* • Overall symptom improvement • Bowel symptom scale, as rated by gastroenterologist	NA NA
		*No difference* • Bowel symptom scale, as rated by participant	NA
	Individualized herbal formulation versus placebo	*No difference* Overall symptom improvement Bowel symptom scale	NA NA
	Herbal medicines versus conventional therapy	65 RCTs assessed 51 different herbal medicines. Data were very heterogenous and not pooled**	NA
	Herbal medicines plus conventional therapy versusconventional therapy alone	9 RCTs assessed herbal medicine in combination with conventional therapy versus conventional therapy alone. Data were very heterogenous and not pooled**	NA
Homeopathy	Asafoetida versusplacebo	*Benefits* • Self-reported overall improvement	Very low
	Asafoetida associated with nux versusplacebo	*No difference* • Self-reported overall improvement	Very low
	Homeopathic consultation plus target treatment versus usual care	*No difference* • Wellbeing outcome	NA
Hypnotherapy	Hypnotherapy versuswaiting list	*Benefits* • Composite primary symptom reduction score • Proportions of hard/watery bowel movements *No difference* • Frequency of bowel motions (12 months) • Proportion of subjects with bloating • Frequencies of bowel motion and abdominal pain	NA NA NA NA NA
	Hypnotherapy plus pharmacological treatment versus pharmacological treatment alone.	*Benefits* • Abdominal pain (3 months) • Composite primary IBS symptom *No difference* • Quality of life (12 months) • Constipation score (3 and 12 months) • Diarrhea score (3 and 12 months) • Overall symptom score (12 months) • Abdominal pain (12 months)	NA NA NA NA NA NA NA
	Hypnotherapy versus psychotherapy plus placebo	*Benefits* • Abdominal pain • Bowel habit • Abdominal distension • General wellbeing	NA NA NA NA
Psychological interventions	Psychological interventions as a groupversususual care	*Benefits* • Symptom score improvement (2 and 3 months) • Abdominal pain improvement (2 and 3 months) • Quality of life (2 months) *No difference* • Quality of life (3 months)	NA NA NA NA
	Psychological interventions as a groupversusplacebo	*Benefits* • Symptom score improvement (2 months) *No difference* • Symptom score improvement (3 months) • Abdominal pain improvement (3 months)	NA NA NA
	Cognitive behavioral therapy versususual care	*Benefits* • Symptom score improvement (3 months) • Quality of life (2 and 3 months) *No difference* • Symptom score improvement (2 months) • Abdominal pain improvement (2 and 3 months)	NA NA NA NA
	Cognitive behavioral therapy versusplacebo	*No difference* • Symptom score improvement (2 and 3 months) • Abdominal pain improvement (2 and 3 months) • Quality of life (3 months)	NA NA NA
	Interpersonal psychotherapy versususual care	*Benefits* • Relief of symptoms *No difference* • Symptom score improvement	NA NA
	Relaxation/stress management versususual care	*Benefits* • Symptom score improvement (2 months) *No difference* • Abdominal pain improvement	NA NA

*GRADE (Grading of Recommendations Assessment, Development and Evaluation) has the aim of assessing the certainty of the body of evidence. From this, the outcomes are classified as having high certainty (high confidence that the estimated effect is close to the true effect); moderate certainty (likely that the estimated effect is close to the real effect, but there is a possibility that it is not); low certainty (limited confidence in the effect estimate) or very low certainty (the true effect is likely to be substantially different from the estimate effect). **For further information about specific types of herbal therapy, refer to the relevant text in the “Results” section of this paper. IBS = irritable bowel syndrome; NA = not assessed; RCTs = randomized clinical trials.

### 1. Acupuncture

It has been hypothesized that acupuncture may have effects on the visceral system through stimulating the somatic system, thereby improving symptoms in patients with IBS. This SR^11^ assessed the effects of acupuncture on IBS and included 17 randomized clinical trials (RCTs) with 1806 participants. Acupuncture was compared with no intervention, sham intervention (placebo for acupuncture) and pharmacological interventions.

#### 1.1. Acupuncture versus sham acupuncture

- Symptom severity: no differences between the groups, as assessed using the IBS severity scoring system^17^ (IBS-SSS), in which lower values are better (standardized mean difference, SMD -0.11; 95% confidence interval, CI -0.35 to 0.13; four RCTs; 281 participants; moderate certainty of evidence).
- Quality of life: no difference between the groups, as assessed using the IBS quality of life scale^18^ (IBS-QOL), in which higher values are better (SMD -0.03; 95% CI -0.27 to 0.22; three RCTs; 253 participants; moderate certainty of evidence).

#### 1.2. Acupuncture versus pharmacological treatment

- Proportion of participants with symptom improvement: higher for acupuncture group (risk ratio, RR 1.28; 95% CI 1.12 to 1.45; 5 RCTs; 449 participants; low certainty of evidence). This outcome was assessed through dichotomization of the scales considered in each RCT, in which a cutoff point was established to decide whether participants had experienced an “improvement”. Likewise, the SR authors found an improvement in this same outcome favoring acupuncture over no specific treatment (RR 2.11; 95% CI 1.18 to 3.79; two RCTs; 118 participants).

Adverse events were reported in nine RCTs. In one RCT, it was reported that one participant had withdrawn due to syncope, while in eight RCTs, no serious adverse events werereported.

The authors of this SR concluded that acupuncture did not provide any benefit for treating IBS patients, compared with sham treatment. Acupuncture seemed to be better than pharmacological interventions or no intervention, but this finding would need to be interpreted with caution and would need to be explored through further RCTs. The fact that the trials were not blinded increased the risk of bias in subjective outcomes such as “symptom improvement”. For further details and to access all the analyses, refer to the original abstract, available from: https://www.cochranelibrary.com/cdsr/doi/10.1002/14651858.CD005111.pub3/full.

### 2. Bulking agents, antispasmodics and antidepressants

This SR^12^ included 56 RCTs (3725 patients) that assessed bulking agents (fiber supplements) (12 RCTs; 621 participants), antispasmodics (29 RCTs; 2333 participants) and antidepressants (15RCTs; 922 participants).

#### 2.1 Bulking agents versus placebo

It was found that bulking agents (including both insoluble and soluble fibers) did not have any beneficial effect in relation to placebo, regarding improvement of abdominal pain (MD 0.03; 95% CI ‐0.34 to 0.40; P = 0.874; 3 RCTs; 186 participants), overall assessment (RR 1.10; 95% CI 0.91 to 1.33; P = 0.32; 11 studies; 565 participants) or symptom score (MD ‐0.00; 95% CI ‐0.43 to 0.43; P = 1.00; 3 RCTs; 126 participants). The subgroup analyses relating to insoluble and soluble fibers were consistent with the main analysis.

#### 2.2 Antispasmodics versus placebo

Antispasmodics had a beneficial effect in relation to placebo for improvement of abdominal pain (58% versus 46%; RR 1.32; 95% CI 1.12 to 1.55; P < 0.001; number needed to treat, NNT 7; 13 studies; 1392 participants), overall assessment (57% versus 39%; RR 1.49; 95% CI 1.25 to 1.77; P < 0.0001; NNT 5; 22 RCTs; 1983 participants) and symptom score (37% versus 22%; RR 1.86; 95% CI 1.26 to 2.76; P < 0.01; NNT 3; 4 RCTs; 586 participants). Subgroup analyses for different types of antispasmodics found that use of cimetropium/dicyclomine, peppermint oil, pinaverium and trimebutine presented statistically significant benefits.

#### 2.3 Antidepressants versus placebo

Antidepressants had a beneficial effect in relation to placebo for improvement of abdominal pain (54% versus 37%; RR 1.49; 95% CI 1.05 to 2.12; P = 0.03; NNT 5; 8 studies; 517 participants), overall assessment (59% versus 39%; RR 1.57; 95% CI 1.23 to 2.00; P< 0.001; NNT 4; 11 RCTs; 750 participants) and symptom score (53% versus 26%; RR 1.99; 95% CI 1.32 to 2.99; P = 0.001; NNT4; 3 RCTs; 159 participants). Subgroup analyses showed that the following presented statistically significant benefits: (a) selective serotonin releasing inhibitors (SSRIs) for improvement of overall assessment; and (b) tricyclic antidepressants (TCAs) for improvement of abdominal pain and symptom score. A separate analysis on studies with adequate allocation concealment found that antidepressants gave rise to significant benefits regarding improvement of symptom scores and overall assessment. Adverse events were not assessed. For further details and to access all the analyses, refer to the original abstract, available from: https://www.cochranelibrary.com/cdsr/doi/10.1002/14651858.CD003460.pub3/full.

### 3. Herbal medicines

Herbal therapies are commonly used for many clinical conditions and it has been hypothesized that these could have benefits for IBS patients. This SR^13^ assessed the effects of herbal medicines on management of IBS and included 75 RCTs (7957 participants). The methodological quality of three RCTs was high, but the overall quality of the remaining RCTs was low. Seventy‐one different herbal medicines were tested alone or in combination with conventional therapy, and were compared with placebo or conventional pharmacological therapy.

#### 3.1 Herbal medicines versus placebo

In 6 RCTs, 12 different herbal medicines were tested in comparison with placebo. Overall, herbal medicines showed better improvement of overall symptoms.

- Standard Chinese herbal formulation
  - Overall symptom improvement: better with herbal formulation, as rated by the participants (RR 2.15; 99% CI1.07 to 4.32) and by the gastroenterologist (RR 2.62; 99% CI1.19 to 5.77).
  - Bowel symptom scale (BSS): no difference between groups after 16 weeks of treatment, as rated by the participants (weighted mean difference, WMD ‐43.90; 99% CI ‐92.16 to 4.36), but better with herbal formulation, as rated by the gastroenterologist (WMD ‐76.30; 99% CI ‐125.45 to ‐27.15). However, this effect was not statistically significant at 14 weeks of follow-up.

- Individualized herbal formulation
  - Overall symptom improvement: no difference between groups (one RCT; 116 participants).
  - Bowel symptom scale (BSS): no difference between groups after 16 weeks of treatment, as rated both by the participants (WMD ‐47.0; 99% CI ‐98.55 to 4.55) and by the gastroenterologist (WMD ‐46.8; 99% CI ‐106.07 to 12.47). Thisfinding was sustained at 14 weeks after completion of the treatment (WMD ‐56.30; 99% CI ‐120.80 to 8.20).

#### 3.2 Herbal medicines versus conventional therapy

In 65 RCTs in which 51 different herbal medicines were tested, 22 herbal medicines resulted in statistically significant symptom improvement and 29 herbal medicines were not significantly different from conventional therapy.

#### 3.3 Herbal medicines combined with conventional therapy versus conventional therapy alone

In nine RCTs in which herbal medicine combined with conventional therapy was evaluated, six showed that there was additional benefit from the combination therapy, compared with conventional monotherapy.

No serious adverse events from the herbal medicines were reported. For further details and to access all the analyses, refer to the original abstract, available from: https://www.cochranelibrary.com/cdsr/doi/10.1002/14651858.CD004116.pub2/full.

### 4. Homeopathy

This SR^14^ assessed the effects of homeopathy for treating IBS patients and included three RCTs (213 participants).

#### 4.1 Asafoetida versus placebo

Asafoetida is a substance derived from the roots of perennial herbs. In two RCTs, use of this substance showed significant benefit regarding the number of patients who had self-reported overall improvements (RR 1.61; 95% CI 1.18 to 2.18; two RCTs; 129participants; very low certainty of evidence).

#### 4.2 Asafoetida associated with nux versus placebo

Nux is a substance derived from seeds that contain strychnine poison. In a single RCT, use of this substance did not show any difference regarding the number of patients who had self-reported overall improvements (RR 1.31; 95% CI 0.80 to 2.15; one RCT; 42 participants; very low certainty of evidence).

#### 4.3 Homeopathic consultation plus target treatment versus usual care

In a single RCT, there was no difference in the wellbeing outcome (MD 0.03; 95% CI -3.16 to 3.22; one RCT; 20 participants).

The very low quality of evidence prevented any solid conclusion about homeopathy for IBS. The RCTs included were small and used non-validated outcomes. Future RCTs with adequate sample size and clinically oriented valid outcomes would need to be performed to reduce the uncertainty in the use of homeopathy for IBS patients. For further details and to access all the analyses, refer to the original abstract, available from: https://www.cochranelibrary.com/cdsr/doi/10.1002/14651858.CD009710.pub2/full.

### 5. Hypnotherapy

Hypnotherapy has been reported to have beneficial effects for managing symptoms. This SR^15^ had the aim of assessing the effects of hypnotherapy for patients with IBS. Four RCTs (147 participants) were included, but no meta-analysis was performed, due to clinical and methodological heterogeneity between the studies.

#### 5.1 Hypnotherapy versus waiting list

Hypnotherapy was superior regarding the composite primary symptom reduction (CPSR) score^19^ (MD -0.87; 95% CI - 1.36 to -0.38) and the proportions of hard/watery bowel movements (MD -0.25; 95% CI -0.38 to -0.12) over the short term, among patients for whom standard medical therapy had failed (37 participants; 2RCTs). Nodifferences between the interventions were found in relation to frequency of bowel motions (12 months), proportion of subjects with bloating, frequency of bowel motion and abdominal pain.

#### 5.2 Hypnotherapy plus pharmacological treatment versus pharmacological treatment alone

Combined therapy was superior regarding abdominal pain after three months (MD -14.4; 95% CI -24.69 to -4.11) and composite primary IBS symptoms (81 participants; one RCT). No differences between the interventions were found in relation to quality of life (after 12 months), constipation score (after 3 and 12months), diarrhea score (after 3 and 12 months), overall symptom score (12 months) and abdominal pain (12 months).

#### 5.3 Hypnotherapy versus psychotherapy plus placebo

There were benefits in the hypnotherapy group at three months in relation to abdominal pain, bowel habit, abdominal distension and general wellbeing (81 participants; one RCT). We entered into correspondence with the authors and found that the data were no longer available for analysis (their study was conducted more than 20 years ago).

No adverse events were reported in any of the trials. Theresults from these studies need to be interpreted with caution due to their poor methodological quality and small size. For further details and to access all the analyses, refer to the original abstract, available from: https://www.cochranelibrary.com/cdsr/doi/10.1002/14651858.CD005110.pub2/full.

### 6. Psychological interventions

Physiological factors appear to be related to one of the pathophysiological aspects of IBS manifestation. There have been some indications of an association between IBS and psychiatric disorders.

The objective of this SR^16^ was to investigate the benefits and harm of any psychological treatments in this population. The SR included 25 RCTs that assessed psychological interventions as a group: cognitive behavioral therapy, interpersonal psychotherapy and relaxation/stress management.

#### 6.1 Psychological interventions as a group versus usual care

- Symptom score improvement: better with psychological interventions at two months (SMD 0.97; 95% CI 0.29 to 1.65; six RCTs; 222 participants) and at three months (SMD 0.62; 95% CI 0.45 to 0.79; eight RCTs; 593 participants), compared with usual care. When compared with placebo, psychological interventions seemed superior at two months (standardized mean difference, SMD 0.71; 95% CI 0.08 to 1.33; two RCTs; 44 participants), but not at three months (SMD ‐0.17; 95% CI ‐0.45 to 0.11; three RCTs; 230 participants).
- Abdominal pain improvement: better with psychological interventions at two months (SMD 0.54; 95% CI 0.10 to 0.98; three RCTs; 90 participants) and at three months (SMD 0.26; 95% CI 0.07 to 0.45; ten RCTs; 727 participants), compared with usual care. In comparison with placebo, no difference was found at three months (SMD 0.31; 95% CI ‐0.16 to 0.79; five RCTs; 416 participants).
- Quality of life: better with psychological interventions at two months (SMD 0.47; 95% CI 0.11 to 0.84; two RCTs; 132 participants), but not at three months (SMD 0.31; 95% CI ‐0.16 to 0.77; three RCTs; 243 participants).

#### 6.2 Cognitive behavioral therapy versus usual care

- Symptom score improvement: no difference through use of cognitive behavioral therapy, compared with usual care at two months (SMD 0.75; 95% CI -0.20 to 1.70; four RCTs; 133 participants). At three months, cognitive behavioral therapy was better than usual care (SMD 0.58; 95% CI 0.36 to 0.79; five RCTs; 378 participants). In comparison with placebo, there was no difference between the groups at two months (SMD 0.68; 95% CI -0.01 to 1.36; two RCTs; 44 participants) and at three months (SMD -0.17; 95% CI -0.45 to 0.11; three RCTs; 230 participants).
- Abdominal pain improvement: no difference through use of cognitive behavioral therapy, compared with usual care at two months (SMD 0.45; 95% CI 0.00 to 0.91; three RCTs; 80 participants) and at three months (SMD 0.22; 95% CI -0.04 to 0.49; seven RCTs; 359 participants). The results were similar in comparison with placebo at two months(SMD -0.41; 95% CI -1.30 to 0.48; one RCT; 20 participants) and three months (SMD 0.33; 95% CI -0.16 to 0.82; five RCTs; 395 participants).
- Quality of life: An improvement through cognitive behavioral therapy was observed in comparison with usual care at two months (SMD 0.44; 95% CI 0.04 to 0.85; two RCTs; 97participants) and at three months (SMD 0.92; 95% CI 0.07 to 1.77; one RCT; 24 participants). In comparison with placebo, no difference was found between the groups at three months (SMD 0.16; 95% CI -0.22 to 0.54; one RCT; 129 participants).

#### 6.3 Interpersonal psychotherapy versus usual care

- Relief of symptoms: better with psychotherapy than with usual care(RR 2.02; 95% CI 1.13 to 3.62; number need to treat, NNT4; two RCTs; 254 participants).
- Symptom score improvement: no difference between the groups (SMD 0.35; 95% CI ‐0.75 to 0.05; two RCTs; 254 participants).

#### 6.4 Relaxation/stress management versus usual care

- Symptom score improvement: better with relaxation/stress group than with usual care at two months (SMD 0.50; 95% CI 0.02 to 0.98; four RCTs; 123 participants).
- Abdominal pain improvement: no difference at three months (SMD 0.02; 95% CI ‐0.56 to 0.61; three RCTs; 158 participants).

Long-term follow‐up results were scarce and there was no convincing evidence that treatment effects were sustained following completion of the treatment, for any treatment type. Forfurther details and to access all the analyses, refer to the original abstract, available from: https://www.cochranelibrary.com/cdsr/doi/10.1002/14651858.CD006442.pub2/full.

## DISCUSSION

This review included six Cochrane systematic reviews (SRs) that evaluated interventions for management of irritable bowel syndrome (IBS). The SRs addressed acupuncture, herbal medicines, homeopathy, hypnotherapy, psychological interventions, bulking agents, antispasmodics and antidepressants. The certainty of evidence ranged from unknown to moderate.

There was moderate certainty of evidence that acupuncture had no important benefit regarding improvement of symptoms and quality of life, compared with sham acupuncture. Additionally, one SR reported with very low certainty of evidence that homeopathic asafoetida, alone or in association with nux, was better than placebo regarding self-reported overall improvement. The other four SRs did not assess the certainty of evidence using the GRADE approach, and therefore future updates need to prioritize this assessment.

Our search strategy also retrieved four Cochrane SR protocols that might be included in a future update of this review.^20^^,^^21^^,^^22^^,^^23^ The aims of these studies are to evaluate probiotic agents for diarrhea‐predominant IBS,^20^ probiotics for IBS in children,^21^ biofeedback^22^ and physical activity.^23^ When published, these SRs will provide the current evidence from these increasingly used interventions for treating IBS and will help guide clinical practice. Also, the present review was restricted to data in the Cochrane Library. However, many SRs have been published by other scientific journals, and these may cover interventions that were not included here.

The fact that the RCTs included in each SR presented methodological and reporting limitations also reduced the certainty of the evidence found. Overall, heterogeneity relating to outcomes and low sample sizes were the most common shortcomings. These,respectively, prevented quantitative synthesis and magnified the imprecision of the findings.

Regarding practical implications, there were no solid conclusions that might reflect a strong recommendation for clinical practice. Healthcare providers and patients need to be aware that there is a lack of evidence from randomized clinical trials to support even the most commonly used interventions for treating IBS. Clinical practice may be individually guided through the results presented in Table 2, but future studies may change these results substantially.

Over the last few years, new classes of drugs have been introduced for management of those patients. However, few RCTs or SRs assessing their effects have been published. Linaclotide,which increases intestinal secretion through activation of guanylate cyclase C, is used for treating constipation and different presentations of diarrhea.^24^ Eluxadoline, a mu-opioid receptor agonist, may likewise be useful for controlling abdominal pain, through regulating gastrointestinal motility, secretions and visceral sensation.^25^ Although few studies have provided any support for a role for special diets in treating IBS, FODMAP diets (based on restriction of fermentable oligosaccharides, disaccharides, monosaccharides and polyols) are frequently used in clinical practice and need to be considered in further studies.^26^ Fecaltransplantation is another controversial topic, and upcoming RCTs and SRs need to encompass assessment of this intervention in future analyses.^27^

 In summary, it is not possible to provide full comprehension of IBS management through addressing only the published SRs. Themajor advances in drugs and alternative treatments that have been published recently make it imperative for updated and GRADE-guided^10^ SRs to be produced. Future RCTs need to focus on the gaps in the evidence and consider clinically relevant outcomes. Core outcome sets need to be developed within IBS research, and trialists should include these in their analyses.

## CONCLUSION

This review included six Cochrane systematic reviews that evaluated acupuncture, herbal medicines, homeopathy, hypnotherapy, psychological interventions, bulking agents, antispasmodics and antidepressants for treating irritable bowel syndrome (IBS). There was moderate certainty of evidence showing that use of acupuncture did not provide any important differences in symptom severity scores and quality of life, in comparison with sham acupuncture. Further well-designed and well-conducted randomized clinical trials are needed in order to reduce the uncertainties regarding several commonly used interventions for treating IBS.
